# CIBZ, a Novel BTB Domain-Containing Protein, Is Involved in Mouse Spinal Cord Injury via Mitochondrial Pathway Independent of p53 Gene

**DOI:** 10.1371/journal.pone.0033156

**Published:** 2012-03-12

**Authors:** Yafei Cai, Jun Li, Shiyong Yang, Ping Li, Xuan Zhang, Honglin Liu

**Affiliations:** 1 College of Life Sciences, Anhui Normal University, Key Laboratory of Biotic Environment and Ecological Safety in Anhui Province, Wuhu, Anhui, China; 2 College of Animal Science and Technology, Nanjing Agricultural University, Nanjing, China; Consejo Superior de Investigaciones Cientificas, Spain

## Abstract

Spinal cord injury (SCI) induces both primary uncontrollable mechanical injury and secondary controllable degeneration, which further results in the activation of cell death cascades that mediate delayed tissue damage. To alleviate its impairments and seek for an effective remedy, mRNA differential display was used to investigate gene mRNA expression profiling in mice following SCI. A specific Zinc finger and BTB domain-containing protein, CIBZ, was discovered to implicate in the SCI process for the first time. Further researches indicated that CIBZ was extensively distributed in various tissues, and the expression level was highest in muscle, followed by spinal cord, large intestine, kidney, spleen, thymus, lung, cerebrum, stomach, ovary and heart, respectively. After injury, the CIBZ expression decreased dramatically and reached the lowest level at 8 h, but it gradually increased to the maximal level at 7 d. Caspase-3 and C-terminal-binding protein (CtBP), two CIBZ-related proteins, showed similar tendency. Interestingly, p53 expression remained constant in all groups. Via flow cytometry (FCM) analysis, it was found that the cell death rate in SCI group markedly increased and reached the highest value 1 d after surgery and the mitochondrial transmembrane potential (ΔΨm) at 1 d was the lowest in all groups. Taken together, it is suggested that: (i) in the presence of CtBP, CIBZ gene is involved in secondary injury process and trigger the activation of apoptotic caspase-3 and bax genes independent of p53; (ii) abrupt down-regulation of CtBP at 8 h is a sign of mitochondria dysfunction and the onset of cell death; (iii) it could be used as an inhibitor or target drug of caspase-3 gene to improve spinal cord function.

## Introduction

Spinal cord injury (SCI) is always an unsolved human challenge. More than half of SCI survivors cannot return to their normal life. Neurological damage after acute SCI results from both primary mechanical injury and subsequent activation of cell death cascades mediating delayed tissue damage [1]–[3]. It is inevitable that the primary injury results from actual mechanical tissue disruption, as well as necrotic cell death. It is controllable that the secondary degeneration results from a cascade of events triggered by the injury, resulting in activation of endogenous cell death pathways [4]. Two morphologically distinct pathways, necrosis and apoptosis, are involved in the secondary process.. The latter is considered as physiological or programmed cell death and may be induced by external or internal stimuli [5]–[9]. The vital role of caspases in apoptosis is well known. The activation of Caspase-3 family proteins has been well demonstrated in animal models, and their inhibitors not only reduced tissue damage, but also improved neurological function [10]. Served as potential target pharmaceuticals, caspase-3 inhibitors have caught the attention of many scientists for curing apoptosis-related diseases and for alleviating impairments induced by SCI [11]–[16]. Many studies have investigated the changes at the RNA and protein levels after spinal cord injury and focused only on a few genes of interest [11], [12], [14], [17], [18], [19]. It is revealed that administration of Ac-DMQD-CHO, a caspase-3 inhibitor, can decrease apoptosis and improve functional outcome in SCI rat model [17]; the broad specific caspase inhibitor (z-VAD fmk) administrated by a variety of routes (including local, subarachnoidal, intravenous and intraperitoneal), following SCI did not improve neurological deficits and tissue damage, or did not prevent XIAP cleavage that is associated with apoptotic death following SCI [13]; in addition, administration of the caspase-3 inhibitor z-DEVD.fmk, a selective and irreversible caspase-3 inhibitor, helped to limit secondary damage and provided significant neuroprotection in rat model [20]. Nimodipine (L-type Ca^2+^ channel antagonist), IL-10 and 2, 2′-methylenebis can alleviate apoptosis-mediated impairments via mitochondrial pathway after spinal cord injury [12], [21], [22]. In summary, it is suggested that agents blocking apoptotic pathways may be of value for treating SCI in the future.

Nevertheless, the foregoing researchers have not reached an agreement in SCI drugs investigation, and the effective materials mentioned above are far away from clinical application practically because none of them is originally screened from SCI animal models. A better understanding of events occurring in the spinal cord after injury is essential to identify ways to limit secondary damage, to promote axonal regeneration, and ultimately to improve functional outcome [23]. For this purpose, we applied mRNA differential display to screen candidate genes related to SCI process from the pool of gene expression profiles. It is discovered that a novel murine BTB domain-containing protein, CIBZ, is involved in spinal cord injury via mitochondrial pathway in mouse model.

## Materials and Methods

### Materials and Grouping

36 mice purchased from Nanjing Qinglongshan Experimental Animal Factory, were reared in separate cages in accordance with the National Institutes of Health guidelines including free diet, 12–12 h light-dark cycles and optimal room temperature [21]. 25–30 g mice were randomly divided into 6 groups (6 mice in each group: 3♂& 3♀): control group underwent sham injuries in which only vertebral plates were cut off without causing any spinal injuries, and the 4 h, 8 h, 1 d, 3 d and 7 d group underwent SCI.

### Animal Model Establishment

In this study, the improved SCI mouse model were established [21]. Animals were anesthetized with chloral hydrate (4 mg/kg, IP). The spinal processes from Th7 to Th9 were exposed in all groups. Mice in 4 h, 8 h, 1 d, 3 d and 7 d SCI groups underwent a 30 g weight drop pressed against the spinal cord for five minutes (30 g×5 min). Successful SCI was confirmed by quick jerks of the hind limbs observed in trauma-surgery animals. All groups were reared under optimal environmental conditions after operation.

### mRNA differential display and SCI-associated gene screening

Mice were euthanized by cervical vertebra dislocation at 4 h, 8 h, 1 d, 3 d and 7 d after SCI, and spinal cord samples were collected. The spinal cords of control group were also sampled at 3 d after sham injuries. Trizol reagent (Invitrogen Co. Ltd.) was used to extract total RNA from the above samples. RT reactions were carried out according to protocol of the kit (Promega Co. Ltd.). Primers combinations between 20 random and 3 anchor primers (20×3 combinations) were used to amplify cDNA samples in the subsequent RT-PCR reactions, and the primer sequences were listed in Table 1 and 2. The PCR products were separated by electrophoresis on 12% polyacrylamide gels (1×TBE buffer, 180 V, 5 h) and stained with AgNO_3_. The differentially expressed fragments were extracted and reamplified by nested PCR, then the products were purified with gel purification kit, cloned into the vector pMD-18T and transformed into E *Coli* DH-5α. Positive clones were sequenced in both directions using an ABI Prism™ 377 (GenScript Corporation, Nanjing, China). The sequences were aligned with BLAST in NCBI and Contig map analysis on the chromosome to decide what kind of gene would be. The differentially expressed bands in the products amplified with primer combination between primer 11 and anchor primer 6 were selected for further research.

**Table 1:** Sequences of random primers.

Labeling of random primer	Primer sequence
1	ACCGCGAAGG
2	GGACCCAACC
3	GTCGCCGTCA
4	TCTGGTGAGG
5	TGAGCGGACA
6	ACCTGAACGG
7	TTGGCACGGG
8	GTGTGCCCCA
9	CTCTGGAGAC
10	GGTCTACACC
11	AGCGCCATTG
12	CACCGTATCC
13	GGGGTGACGA
14	CTTCCCCAAG
15	CATCCGTGCT
16	AGGGCGTAAG
17	TTTCCCACGG
18	GAGAGCCAAC
19	GAGAGCCAAC
20	ACCCGGTCAC

**Table 2:** Anchorage primer sequences.

Labeling of anchorage primer	Primer sequence
H-T11G (anchorage4)	AAGCTTTTTTTTTTTG
H-T11C (anchorage 5)	AAGCTTTTTTTTTTTC
H-T11A (anchorage 6)	AAGCTTTTTTTTTTTA

### CIBZ mRNA expression in various tissues by RT-PCR

Total RNA was extracted from various tissues (including kidney, stomach, thymus, large intestine, lung, spinal cord, spleen, heart, muscle, ovary and cerebrum etc.). RT-PCR was processed according to section 1.3. A pair of primers was redesigned to amplify CIBZ gene (listed in Tab.3). A pair of primers (upstream: 5′-GCAATGCCTGGGTACATGGTGG-3′; downstream: 5′-GTCGTACCACAGGCATTGTGATGG-3′) was used to amplify the β-actin gene as an internal standard. PCR products were separated on 2% agarose gel and stained with ethidium bromide. To confirm the identity of PCR products, single bands of the expected size were excised from the gels and sequenced. The CIBZ gene expression levels in different tissues were calculated with BandScan v5.0 software.

### CIBZ expression and distribution

CIBZ was also detected immunohistochemically in the spinal cord after injury. CIBZ antibodies (NBP1-18299, Novus Biologicals Co. Ltd., USA) were diluted in antibody diluent according to ABC kits (Boster, Wuhan, China) in advance. Th7–Th9 tissue samples fixed in phosphate-buffered 10% formalin and embedded in paraffin wax were serially sectioned (5 µm), and mounted on APES-coated slides (Boster, Wuhan, China). These slides were dewaxed, washed in citrate buffer (pH 6.0; 10 min), blocked in 10% normal goat serum/4% BSA (overnight at 4°C) and then incubated (1 h at room temperature) with CIBZ antibody. After rinsing in 0.05 ml PBS, sections were incubated for 1 h in biotinylated sheep anti-mouse IgG antibodies diluted 1∶500 antibody diluent. After washing with PBS and subsequent incubation for 1 h, the Streptavidin peroxidase complex was applied. After washing again, the peroxidase reaction was carried out in DAB solution containing 0.01% of H_2_O_2_ in Tris-HCl buffer (50 mM, pH 7.6) and then washed with distilled water again. After light counterstaining with Mayer's hematoxylin, the sections were dehydrated and covered with cover slip. Photomicrographs were taken with a Leica DM 2500 microscope. The dark-brown positive cells in sections were counted using 10× ocular and 40×objective (400×). The average number of positive cells from 20 randomly-selected fields of different groups were determined and used in data analysis. The integrate optic densities (IOD) of CIBZ immune positive (IR) neural fibers and neurons were analyzed by *Image-Pro Plus* 6.0 (IPP6.0).

### CIBZ expression after SCI injury by Western-blot analysis

Spinal segments Th7–Th9 were collected from animals in all groups and digested by 0.25% trypsin–0.02% EDTA (Gibco Laboratories, USA). After suspension, the dissociated cells were rinsed with PBS and filtered with nylon net (400 holes). Cells suspension (3×10^6^ for each sample) (from 1.3) was centrifuged at 3000 rpm for 5 min at 4°C, then the Cells precipitation was washed with PBS and suspended in extraction buffer and lysis solution. For analysis of CIBZ, 25 µg extracted proteins were separated on a 12% SDS-PAGE denaturing gel and transferred to nitrocellulose membrane (Amersham BioSciences, Piscataway, NJ). Equivalent loading was checked by actin staining. Membranes were blocked in 5% milk then probed with CIBZ antibodies (1∶200). Expression of proteins was quantified by density analysis using BandScan v5.0 software.

### CIBZ-pathway associated gene expression by relative RT-PCR

The cDNA samples collected at different phases were used to analyze the CIBZ-pathway associated gene expression level. With β-actin being an internal standard, p53, caspase-3, CIBZ and CtBP gene expression were studied using semi-quantitative RT-PCR. The primers were designed and confirmed to be unique in the nonredundant NCBI Database and were listed in Table 3. CIBZ and β-actin primer sequences were the same as before. The reaction products and their intensities in RT-PCR were quantified densitometrically and normalized for the expression of the β-actin gene using BandScan v5.0 software.

**Table 3:** Targe genes, GenBank accessions, primer sequences, predicted size of PCR products and annealing temperatures in RT-PCR analysis.

Target gene	GenBank accession	Primer sequence	Size of predicted product	Annealing temperature
CIBZ	NT-039476	Upstream: 5′-GCTCGTGCTTCTGTAGGTTC-3′Downstream:5′-GCTTTGCCAGGCTGAATGTGT-3	500 bp	55.9°C
caspase-3	NM-009810	Upstream: 5′-AGCTTCTTCAGAGGCGACTA-3′Downstream:5′-GGACACAATACACGGGATCT-3′	381 bp	58.5°C
CtBP	NC-000081	Upstream: 5′- ACAGGTACAGCACAGGCA -3′Downstream: 5′- TGCTGAACAGAGAAAGGA -3′	630 bp	56.7°C
p53	NT-033777	Upstream: 5′- ACTGCATGGACGATCTGTTG -3′Downstream:5′-GCCATAGTTGCCCTGGTAAG -3′	495 bp	58.8°C

### The cell death rate in the spinal cord was analyzed by flow cytometry after SCI injury

Cells from animals in all groups were diluted into (1–5)×10^6^ cell/ml, fixed with 70% ethanol (4°C) for 1–2 h and rinsed with PBS (5 min×3). The cells were analyzed with flow cytometry (Becton Dickinson) after being stained with 1 ml propidium iodide (including RNAase) for 30 min. The wavelength of laser was 488 nm and the wavelength of emitted light was above 630 nm. The histogram was used to assay the red intensity of propidium iodide. The software Cell Quest and Modfit LF were used to assay the cell death rate of 10000 cells. The number of cell death was counted and expressed with blue wave peak [21].

### Measurement of mitochondrial transmembrane potential (ΔΨm) of sipial cord cells by flow cytometry

Flow cytometric analysis of mitochondrial activity was determined by using rhodamine 123 (Rh123). Loss of mitochondrial transmembrane potential (ΔΨm) results in diminished cell ability to accumulate fluorochrome Rh123. The suspended spinal cord cells were then incubated with 10 mg/ml Rh123 at 37°C for 30 min, digested by 0.25% trypsin-0.02% EDTA and washed with ice-cold PBS. Finally, the cells were washed and re-suspended in 0.5 ml PBS before fluorescence was measured using flow cytometry [21].

### Statistic Analysis

The data were expressed as mean ± SD and analyzed using one-way ANOVA (SPSS 17.0 software package). LSD-*t* test was used for multiple comparison to identify differences between groups of 5% and 1% significance level. Original data were Z-score transformed as needed to ensure homogeneity of variance.

## Results

### CIBZ was detected in mRNA differential display analysis

**Figure 1:**
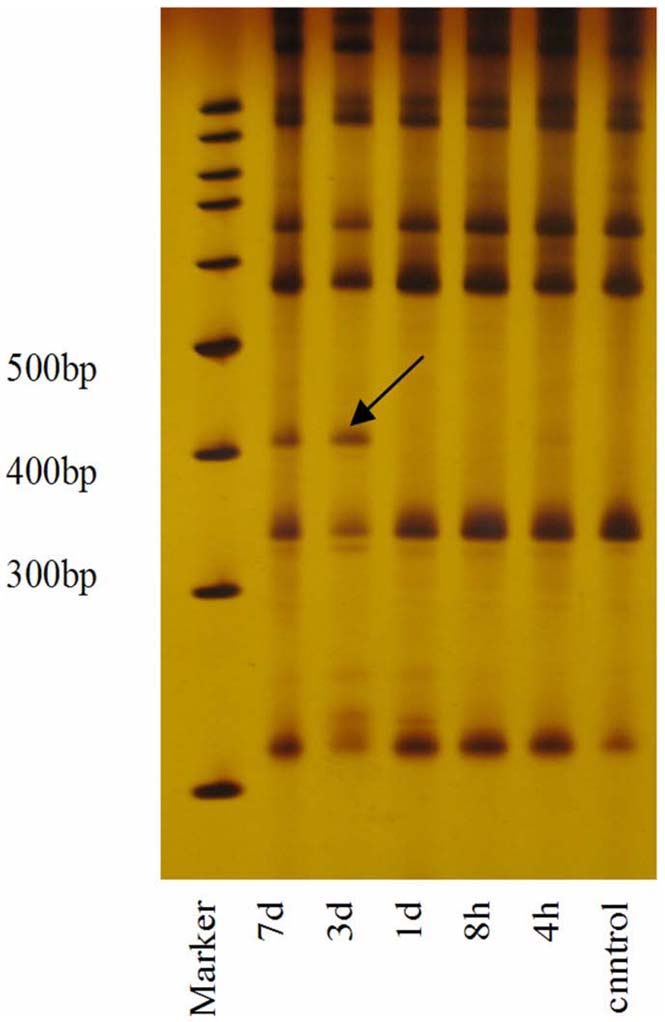
mRNA profiles of different groups upon DDRT-PCR. Black arrow: A differential expression band in this study amplified with primer 11 and anchorage 6. Different lanes respectively represented: control, 4 h, 8 h, 1 d, 3 d and 7 d groups.

A 430 bp differential expression band amplified with combination between primer 11 and anchor primer 6 was detected in expression profiles (black arrow, Figure 1). The band was excised, extracted and amplified with nested PCR (Figure S1a). The PCR product was sequenced (Figure S1b).

The sequence was aligned with BLAST in NCBI (Figure S2a), and the sequence analysis of the cDNA revealed that it is 99% homologous to mouse 16274888–16275218 bp sequence on the 9^th^ chromosome. BLAST hits indicated that it was exactly located at the 16257627–16303924 bp sites of ref|NT_039476.7|Mm9_39516_37 on the 9^th^ chromosome. The differential expression fragment belonged to 5′-UTR of mouse CIBZ (ZBTB38) gene through Contig map analysis on the chromosome (Figure S2b).

### CIBZ mRNA expression in various tissues *in vivo*

**Figure 2:**
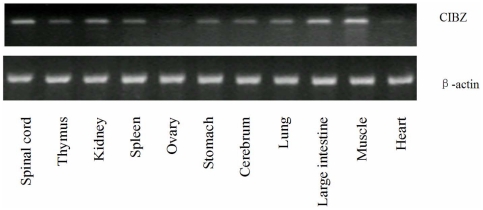
The electrophoresis photo and CIBZ expression of different tissues upon RT-PCR analysis. CIBZ gene was widely distributed in different tissues, especially in spinal cord.

The size of amplified CIBZ fragments was the same as expected (500 bp). CIBZ gene is extensively distributed in various tissues and no tissue-specific expression was observed. The expression level was highest in muscle, followed by spinal cord, large intestine, kidney, spleen, thymus, lung, cerebrum, stomach, ovary and heart etc, respectivley (Figure 2).

### IOD of CIBZ immune positive (IR) neural fibers and neurons after SCI injury

**Figure 3:**
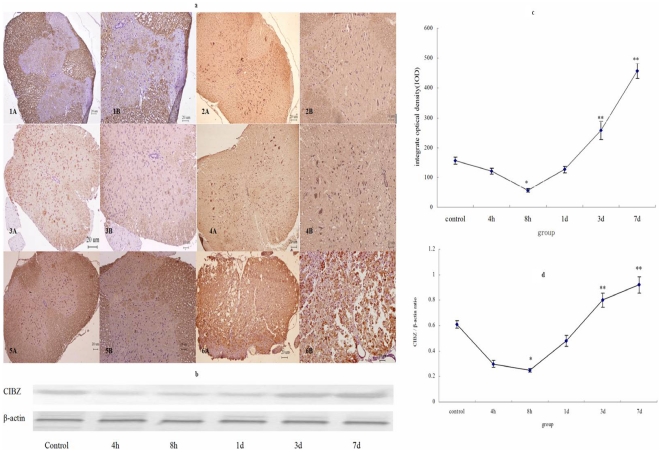
CIBZ protein expression level in the spinal cord after SCI injury. a: CIBZ immunohistochemistry staining in spinal cord after SCI injury. 1A, 1B: control group; 2A, 2B: 4 h group; 3A, 3B: 8 h group; 4A, 4B: 1 d group; 5A,5B: 3 d group; 6A, 6B: 7 d group. Positive cells were observed in both gray and white matter, and the staining in the white matter was much more intense than those in other regions. c: Statistic analysis of CIBZ-IR IOD. CIBZ-IR IOD decreased at first and up-regulated later, the inflection point was at 8 h. Data are expressed as mean±SD. *, *P*<0.05; **, *P*<0.01; N = 6. b: CIBZ expression after SCI injury subjected to Western-blot analysis. The immunoblot result indicated that CIBZ level increased slightly at 4 h, declined sharply at 8 h and gradually increased from 1 d to 7 d. d: Statistic analysis of CIBZ protein level. Data are expressed as mean±SD. *, *P*<0.05; **, *P*<0.01; N = 6.

Immunohistochemical staining identified that the cytoplasm of all positive cells were stained in yellow-brown; positive cells were observed in both gray and white matter; staining in the white matter was much more intense than that in other regions (Figure 3a); In addition, the IOD of CIBZ-IR neural fibers and neurons in control group decreased immediately after SCI injury, then reached the lowest (56.7±6.8) level at 8 h. However, it dramatically increased and reached the highest level (457.2±25.6) at 7 d after injury (Figure 3c). Statistical analysis revealed that there was significant difference in the IOD levels between the sham-operated control groups and SCI groups (*P*<0.01). In the 7 d group, it was also found that the spinal cord presented vacuolization, neurons decreased and glial cells teemed (Figure 3a).

### CIBZ protein level following injury

CIBZ level of spinal cord decreased accordingly after SCI injury, It reached the lowest level at 8 h, and then upregulated and reached the highest at 7 d following injury (Figure 3b, 3d). The CIBZ variation almost had the same tendency as IOD change.

### CIBZ, caspase-3, CtBP and p53 gene expression in spinal cord of different groups

**Figure 4:**
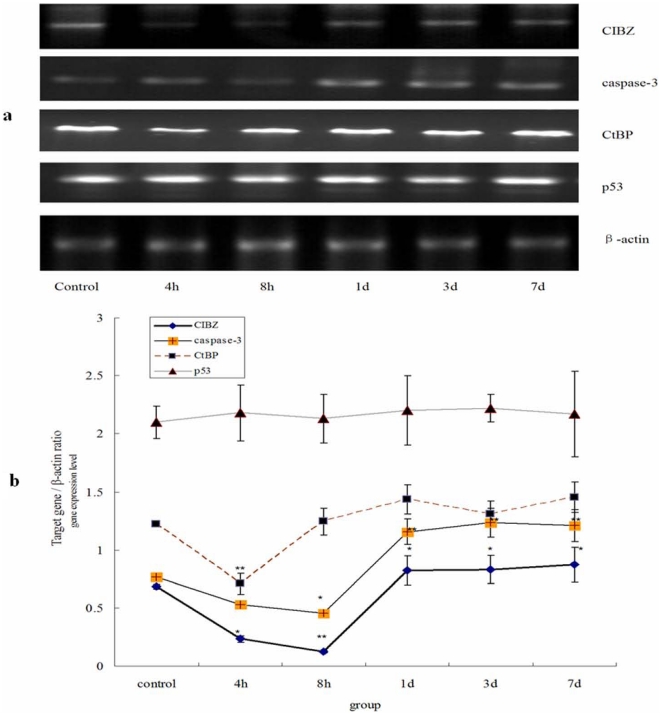
Analysis of CIBZ, caspase-3, CtBP and p53 mRNA level by semi-quantitative RT-PCR. a: The expression level of target gene mRNA analyzed by semi-quantitative RT-PCR. b: Statistic analysis of CIBZ, caspase-3, CtBP and p53 mRNA level. CIBZ and caspase-3 reached the lowest level at 8 h, but CtBP at 4 h. p53 remains constant in all groups. Data are expressed as mean±SD. *, *P*<0.05; **, *P*<0.01; N = 6.

4–8 h after injury, CIBZ gene expression declined drastically to between 20–50%,,then gradually increased from 8 h to 7 d (Figure 4a, 4b). CIBZ expression was lowest at 8 h, but it rebounded to the peak at 7 d. And there was significant difference between the levels at 4 h, 8 h, 1 d, 3 d, 7 d and the control groups after injury (*P*<0.05). Caspase-3 gene also decreased from 4 h to 8 h after injury, then increased from 1 d to 7 d. Caspase-3 expression was the highest at 3 d, and there was significant difference between the levels at 1 d, 3 d, 7 d and the control groups after injury (*P*<0.01). CtBP expression suddenly decreased at 4 h, then restored to normal level, and there was significant difference between the levels for the control, 8 h, 1 d, 3 d, 7 d and 4 h groups after injury (*P*<0.01). Interestingly, p53 expression remained constant in all groups (Figure 4a, 4b).

### The changes of cell death rate after SCI

**Figure 5:**
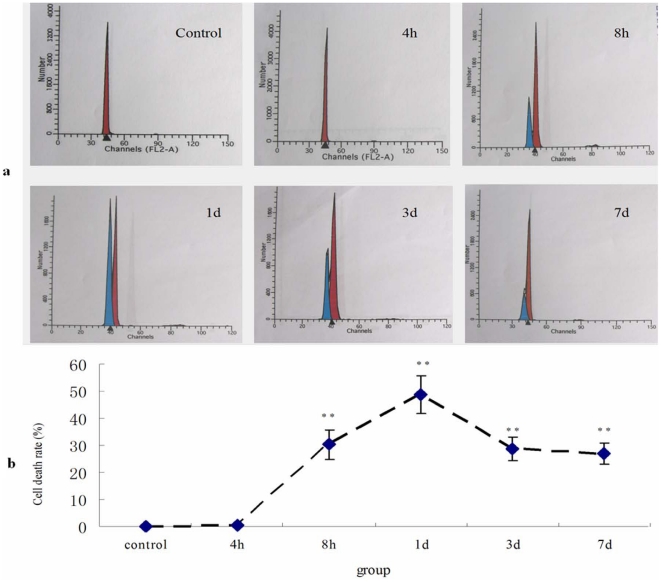
The cell death rate in all groups by FCM analysis. a: FCM analysis of all groups. b: Statistic analysis of cell death rate. The data indicated that the rate ascended rapidly after 4 h post injury, arrived at the highest level at 1 d and decreased subsequently and remained about 30% level. Data are expressed as mean±SD. *, *P*<0.05; **, *P*<0.01; N = 6.

The cell death rate (Figure 5a) in SCI group increased profoundly from 0.34% at 4 h to 30.24% at 8 h, and rapidly reached the highest (48.73%) at 1 d after surgery (Figure 5b), but it decreased to 28.74% at 3 d and 27.02% at 7 d. It is significantly different from the control and 4 h groups (Figure 5b) (P<0.01).

### Mitochondrial membrane potential (ΔΨm) changes of spinal cord cells after SCI

**Figure 6:**
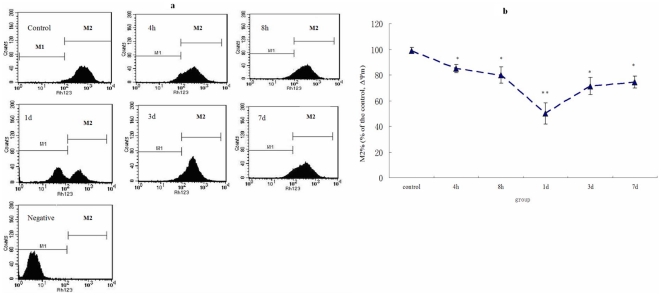
Mitochondrial membrane potential (ΔΨm) changes after spinal cord injury by FCM analysis. a: Histograms of all groups by FCM. b: Statistic analysis of ΔΨm. If fluorescence intensity decreased, the ΔΨm and the ability of the cells to accumulate RH123 decreased accordingly. M2% gates as a percent of the control, which reflected the ΔΨm in all groups, were also shown in line graph. ΔΨm at 1 d is the lowest in all groups. Data are expressed as mean±SD. *, *P*<0.05; **, *P*<0.01; N = 6.

The mitochondrial transmembrane potential decreased immediately and reached its lowest value at 1 d after SCI, and it was significantly different from the control group (P<0.01). ΔΨm gradually increased from 1 d to 7 d afterwards (Figure 6a, 6b). The cellular ability to uptake Rh123 underwent dramatic reduction after SCI.

## Discussion

### CIBZ is involved in SCI process independent of p53 gene

For the first time, we found that CIBZ gene was involved in SCI process *in vivo* in the present study. CIBZ (ZBTB38 in human) is one of the members of BTB domain-containing protein family containing Kaiso-like zinc finger, however, little is known about its physiological function [24], [25]. CIBZ interacts with CtBP and binds methylated DNA, so it belongs to a novel class of methylated DNA-binding proteins [26], [27], [28]. It was reported that CIBZ was highly expressed in proliferating C2C12 cells but its expression levels decreased upon induction of apoptosis by serum starvation. Knockdown of CIBZ in C2C12 cells induced apoptosis, activation of caspase-3, 7, 9, and cleavage of poly (ADP-ribose) polymerase [18], [27], [28], [29]. It is suggested that CIBZ-associated apoptosis occurs through the mitochondrial pathway *in vitro*. CIBZ^−/−^p53^−/−^mouse embryonic fibroblast cells also activated caspase-3 [26], [27]. It can be inferred that CIBZ-associated apoptosis is mediated by a p53-independent pathway *in vitro*.

However, the foregoing studies were based on the evidences *in vitro*. In this study, it was found that CIBZ declined after injury, then increased suddenly from 1 d. It was previously reported that the proportion of early apoptotic cells and late cell death increased significantly to their maximum levels from 1 d to 3 d after SCI via FCM analysis [21], [30]. Nevertheless, the minimum value of CIBZ expression occurred at 8 h. It was also puzzling that caspase-3 gene expression was not contrary to CIBZ expression as expected. The conditions *in vivo* are completely different from those *in vitro*, so we suggest that injury-induced down-regulated CIBZ expression is an early event *in vivo*, while apoptosis and caspase-3 family activation were later events. Moreover, seen from almost unchanged p53 expression, we could deduce that CIBZ gene was also involved in SCI independent of p53 *in vivo*.

CIBZ expression increased from 1 d to 7 d, suggesting that spinal shock induced compensatory mechanism in improving the CIBZ expression for self-healing. Hence, it is beneficial and effective for animal recovery at later stage as high level CIBZ can suppress ill effects induced by high level of caspase-3. In previous studies, it was found that c-kit gene up-regulated and reached peak value from 8 h to 7 d. As a marker of stem cell, its up-regulation signified the activation of stem cells and rehabilitation improvement [30], [31], [32]. Whether the two factors cooperate and promote the process needs our further investigation on the underlying sense.

In conclusion, as a reported negative regulator of apoptosis induced by capase-3 mitochondrial pathway in murine cells [27] *in vitro* and a promising SCI medication, CIBZ potentially alleviates secondary injury *in vivo*. Providing CIBZ is medicated in early injury period (4 h–8 h) or used genetically through CIBZ modified neural stem cells (NSCs) [33], it would deter the cell death-induced impairments. Further researches are needed to explore this possibility.

### Sudden CtBP down-regulation at 8 h is a sign of mitochondria dysfunction and the onset of cell death

CtBP expression drastically declined to a minimum at 4 h, then restored to the normal level. It is reported that CtBP-knockout cells were hypersensitive to apoptosis. CtBP-rescued mouse embryo fibroblasts revealed that CtBP regulates many pro-apoptotic genes and modulates the cellular threshold for apoptotic responses. CtBP can repress Bcl-2-associated X protein (Bax) transcription [34]. CtBP knockout increases Bax transcription, ablates mitochondrial morphology and reduces mitochondrial activities. Ectopic expression of CtBP or knockdown of Bax in CtBP-knockout cells recovers mitochondrial morphology and function, suggesting that CtBP functions as a metabolic sensor that maintains mitochondrial activities *in vitro* [35]. In previous studies, it was found that bax regulation from 4 h and injury-induced mitochondria dysfunction after SCI [30], [31], [32]. In this study, the mitochondrial transmembrane potential reduced to the lowest level at 24 h. It can be inferred that sudden CtBP down-regulation at early stage is also a prelude of mitochondria dysfunction and onset of cell death *in vivo*.

Besides, Several reports linking CtBP with p53-independent cell death *in vitro* [27], [36], [37] : (i) targeting of CtBP by ARF (alternate open reading frame) results in p53-independent apoptosis; (ii) HIPK2 (homeodomain interacting protein kinase 2) mediates CtBP phosphorylation and degradation in UV-induced cell death; (iii) UV triggered active JNK1 (c-Jun N-terminal kinase 1) promotes apoptosis by phosphorylated and down-regulated CtBP in human lung cancer cells. These findings demonstrated that CtBP protein plays important roles in regulating p53-independent programmed cell death, although the precise mechanism and signaling pathway remain unclear. In the present study, unchanged p53 expression and CtBP down-regulation do indicate that CtBP is involved in spinal cord injury independent of p53 *in vivo*.

In summary, in the presence of CtBP, CIBZ gene is involved in secondary injury process. CIBZ possibly triggers the activation of apoptotic caspase-3 and bax genes independent of p53, and cell death-induced jury burst. It is suggested that CIBZ is able to be used as an inhibitor or target drug of caspase-3 gene in improving spinal cord function.

## Supporting Information

Figure S1 **Identification of CIBZ gene.** S1a: The differential expression fragment (black arrow) was re-amplified with nested PCR. The size of PCR products was 430 bp. S1b: Sequencing wave of CIBZ gene. Sequencing result of differential expression fragment was also shown.
(TIF)

Figure S2 **The analysis of differential expression fragment.** S2a: Differential expression sequence was aligned with BLAST in NCBI. It is 99% homologous to mouse 16274888–16275218 bp sequence on the mouse 9th chromosome. S2b: Contig map analysis indicated that it belongs to 5′-UTR of CIBZ gene.
(TIF)
