# Supplementary figures and images for: CIBZ, a Novel BTB Domain-Containing Protein, Is Involved in Mouse Spinal Cord Injury via Mitochondrial Pathway Independent of p53 Gene
Source: PLoS One. 2012 Mar 12;7(3):e33156. doi: 10.1371/journal.pone.0033156 (PMC3299754; doi:10.1371/journal.pone.0033156)

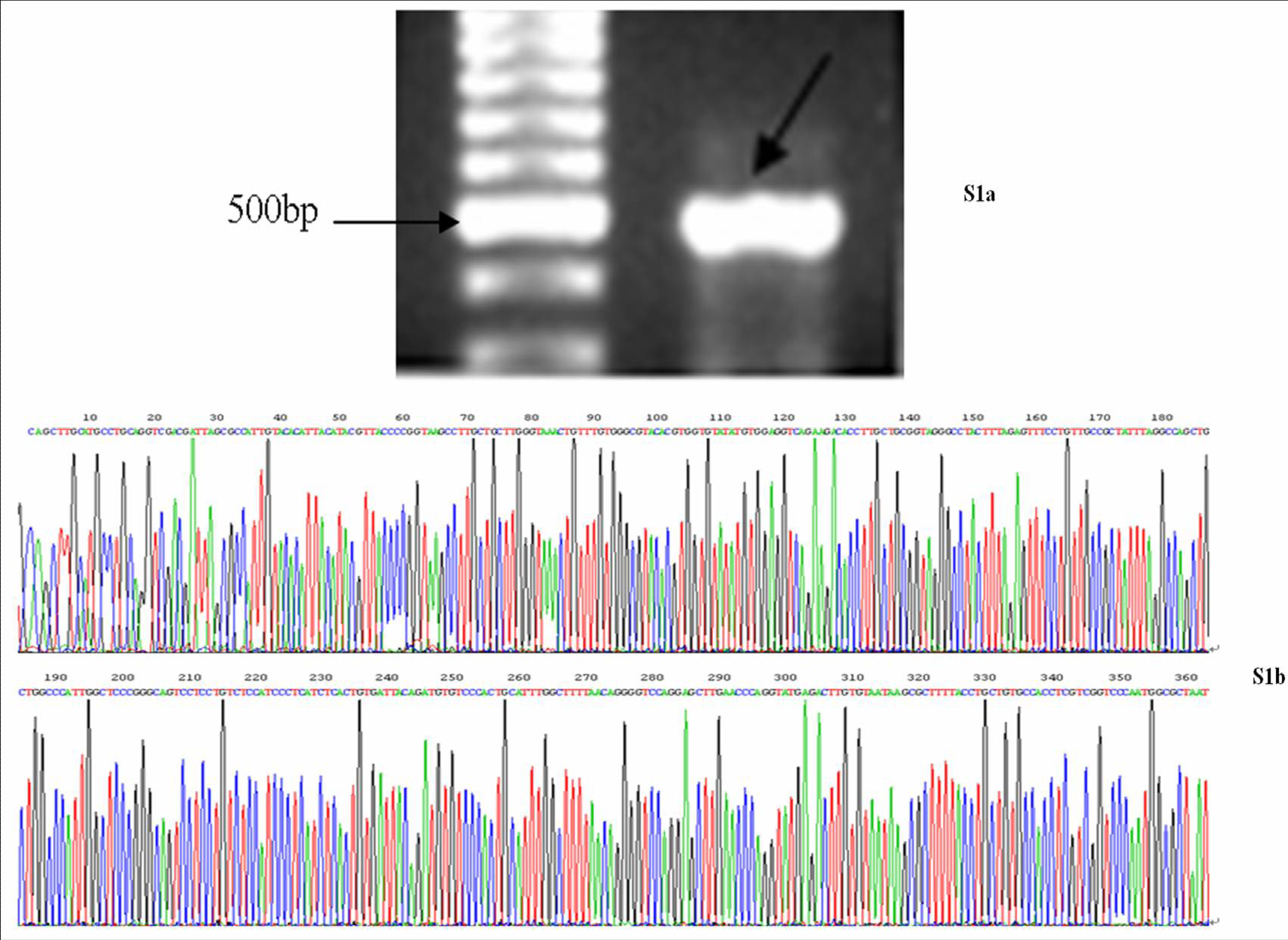

Supplement: Figure S1 — Identification of CIBZ gene. S1a: The differential expression fragment (black arrow) was re-amplified with nested PCR. The size of PCR products was 430 bp. S1b: Sequencing wave of CIBZ gene. Sequencing result of differential expression fragment was also shown. (TIF) [file pone.0033156.s001.tif]

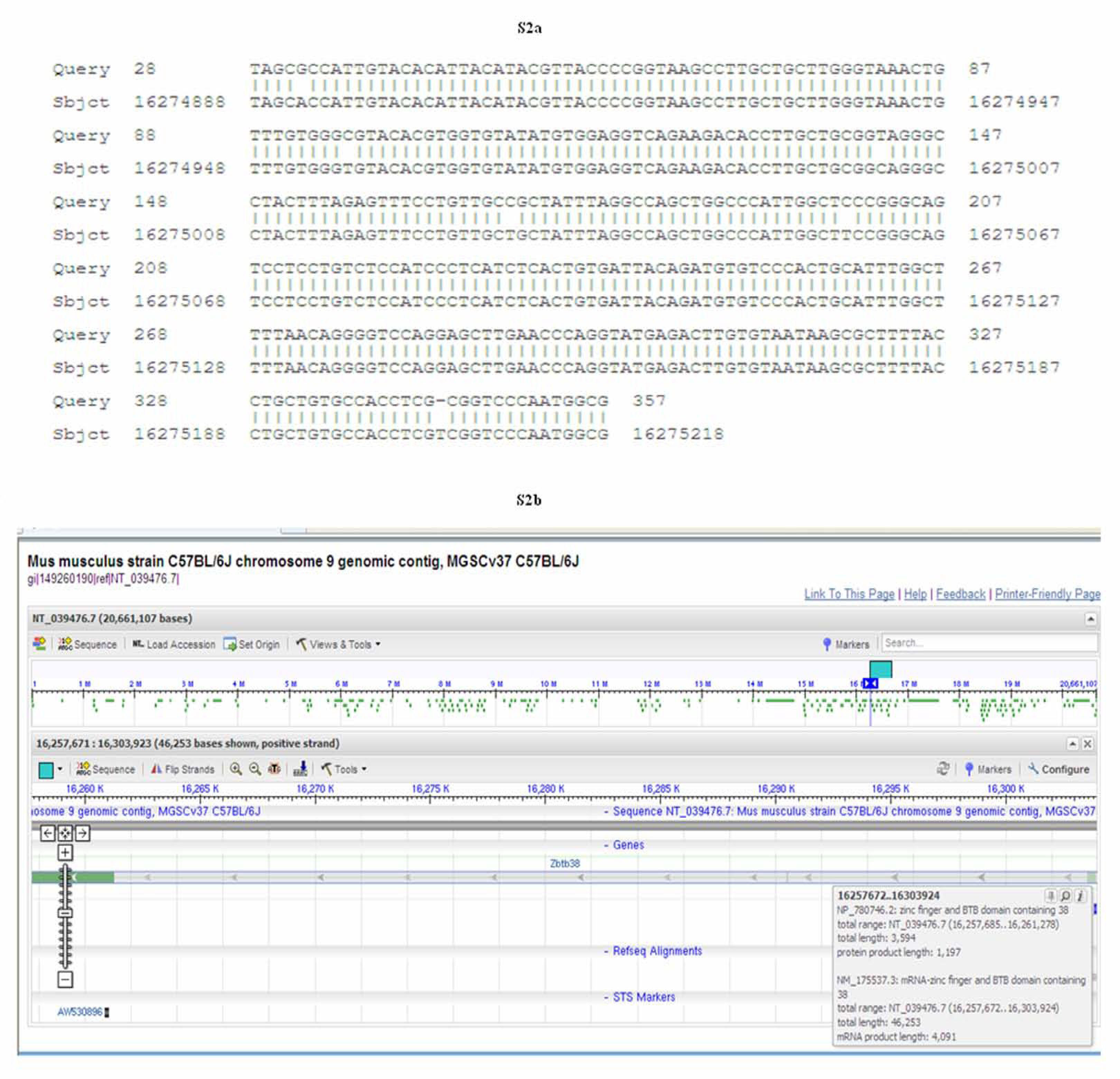

Supplement: Figure S2 — The analysis of differential expression fragment. S2a: Differential expression sequence was aligned with BLAST in NCBI. It is 99% homologous to mouse 16274888–16275218 bp sequence on the mouse 9th chromosome. S2b: Contig map analysis indicated that it belongs to 5′-UTR of CIBZ gene. (TIF) [file pone.0033156.s002.tif]
